# The Awareness, Prevalence, and Risk Factors of Chronic Kidney Disease Among Diabetes Mellitus and Hypertensive Patients in the Aseer Region, Saudi Arabia

**DOI:** 10.7759/cureus.53366

**Published:** 2024-02-01

**Authors:** Mohammed Al-qahtani, Ibrahim Tawhari, Abdulaziz M Alhmare, Abdullah S Badawi, Abdullah Alsalem, Mohammed A Gazzan, Adel M Hamdi, Abdullah Rashid, Ali M Alqahtani

**Affiliations:** 1 Medicine, College of Medicine, King Khalid University, Abha, SAU; 2 Internal Medicine, College of Medicine, King Khalid University, Abha, SAU; 3 Medicine, College of Medicine, King Khalid University, Khamis Mushait, SAU

**Keywords:** saudi arabia, attitude, knowledge, prevalence, hypertension, diabetes, chronic kidney disease

## Abstract

Background and objective

Given its ever-increasing burden, chronic kidney disease (CKD) represents a significant public health threat. CKD is characterized by a gradual alteration in the kidney's normal glomerular filtration rate, which results in the progressive loss of kidney function over a period of time ranging from a few months to years. Diabetes mellitus (DM) and hypertension (HTN) are well-known risk factors for developing CKD and end-stage renal failure. In light of this, this study aimed to assess the awareness, prevalence, and risk factors of CKD in patients with diabetes and those with HTN in the Aseer region, the Kingdom of Saudi Arabia.

Methods

A correlational cross-sectional study was conducted among a sample of people across Saudi Arabia. The data collection was conducted via an online questionnaire circulated on social media platforms. The study questionnaire included socioeconomic and demographic information and medical history of DM, HTN, and CKD. Also, patients' awareness of and attitude towards CKD were assessed.

Results

A total of 301 diabetic or hypertensive patients fulfilling the inclusion criteria completed the study questionnaire. Of them, 174 (57.8%) were aged less than 55 years, while 127 (42.2%) were aged more than 55 years; 200 (66.4%) patients were males. A total of 94 (31.2%) study patients were diabetic, 64 (21.3%) were hypertensive, and 143 (47.5%) were both diabetic and hypertensive; 226 (75.1%) study patients had an overall good awareness of CKD while only 75 (24.9%) showed a poor awareness level. Higher awareness was associated with patients' age, education, and having CKD, DM, or HTN (p<0.05).

Conclusion

Our findings revealed that CKD was not common among study patients, and its prevalence was found to be less than estimated based on many studies in the literature. Also, diabetic and hypertensive patients showed a higher than satisfactory level of awareness of CKD, especially young patients with high levels of education.

## Introduction

Chronic kidney disease (CKD) is a condition that leads to progressive deterioration in kidney function over an extended period, typically spanning months to years [1]. It is characterized by the gradual substitution of the healthy structure of the kidneys with fibrous or scarred tissue, impairing their ability to perform their essential functions, thereby reducing the kidneys' capacity to filter blood wastes while also carrying out other tasks. End-stage kidney disease results in kidney failure [2-3]. CKD is considered a significant global health problem, and it represents a great burden worldwide on account of its complications [4-5]. Globally, in terms of mortality, morbidity, and risks related to the heart and blood vessels, CKD has a direct impact on the global burden. The prevalence of diabetes mellitus (DM) and high blood pressure, in addition to obesity and aging, are some of the causes of the global burden associated with CKD [6-7]. There are 4.902 million to 7.083 million patients with end-stage kidney disease who require renal replacement therapy [8].

More than 20,000 CKD patients in the Kingdom of Saudi Arabia are currently on dialysis, and 9,800 patients are undergoing follow-up after kidney transplantation. It is estimated that the combined incidence of kidney replacement therapy in Saudi Arabia is about 294.3 per million people [9]. Due to the increased hazards that accompany poverty, such as infection, inadequate education, dangerous job conditions, and higher direct expenditures for evaluation and treatment, CKD is more prevalent in developing countries [10-11]. CKD in developed countries is commonly attributed to diabetes, in addition to high blood pressure. Despite this well-documented association, studies have shown that less than 5% of patients who suffer from CKD at an early stage are aware of their illness in the areas where these studies were conducted [12]. Early detection of CKD and its prompt treatment can lead to the prevention and reduction of complications associated with the condition. Cases of CKD are generally non-clinical, but mainly occur due to a lack of awareness among patients about kidney diseases, chronic diseases, and factors associated with them [13]. The current study aimed to assess the awareness, prevalence, and risk factors of CKD among patients with diabetes and those with hypertension (HTN) in the Aseer region of Saudi Arabia.

## Materials and methods

Study design, setting, and participants

A descriptive, cross-sectional, web-based study was conducted to assess diabetic and hypertensive patients' awareness regarding CKD in the Aseer region, Saudi Arabia. All diabetic or hypertensive patients aged 18 years or older who consented to participate in the study were enrolled in the study’s final analysis. Healthy respondents, those aged less than 18 years, and those living outside the Aseer region were excluded. An online questionnaire was developed by the researchers based on a literature review and consultation with the field experts. The validity of the questionnaire and its applicability and level of clarity were assessed by three expert staff independently, and necessary modifications were made until the final version of the used questionnaire was created.

The anonymous questionnaire was published on various social media platforms from 20/7/2023 to 30/9/2023. All eligible patients were encouraged to participate in this study by ensuring the confidentiality of their participation and the importance of this research to the general society's health. The questionnaire for this study included participants' demographic data (age, gender, geographic location, education, and monthly income). Also, patients' awareness regarding CKD was assessed using closed-ended questions with one correct answer. Part 3 of the questionnaire covered patients' attitudes toward CKD using closed-ended questions with "agree" or "disagree" options. The final validated questionnaire was uploaded online on social media platforms by the researchers and their associates to be accessed by eligible patients.

Data analysis

The data underwent a series of steps, including collection and review, before being input into SPSS Statistics version 21 for analysis (IBM Corp., Armonk, NY). Statistical analyses were conducted using two-tailed tests with an alpha level of 0.05, and the results were deemed significant if the p-value was less than or equal to 0.05. To assess the overall awareness level toward CKD, scores for different items related to awareness were summed up. If a patient's score was less than 60% of the overall score, their awareness level was categorized as poor. Conversely, if their score was 60% or higher, their awareness level was classified as good. 

The study variables, including participants' personal data, medical history, and family history of CKD, HTN, and DM were analyzed using descriptive statistics. These data were presented as frequency and percentage. The awareness levels regarding CKD were tabulated, and the overall awareness was graphically depicted. To identify factors associated with patients' awareness of CKD, cross-tabulation was performed. This involved using the Pearson chi-square test for significance, and an exact probability test was used if there were small frequency distributions.

## Results

A total of 301 diabetic and hypertensive patients who fulfilled the inclusion criteria completed the study questionnaire. Of them, 174 (57.8%) were aged less than 55 years, while 127 (42.2%) were aged more than 55 years; 200 (66.4%) patients were males, and 101 (33.6%) were females. As for educational level, 64 (21.3%) had a low educational level (below high school), 92 (30.6%) had a high school education, and 145 (48.2%) had a university level of education or above (Table 1).

**Table 1:** Demographic characteristics of the study participants (N=301)

Variables	Frequency (n)	Percent (%)
Age in years		
<55	174	57.8%
>55	127	42.2%
Gender		
Male	200	66.4%
Female	101	33.6%
Educational level		
Below high school	64	21.3%
High school	92	30.6%
University/above	145	48.2%

Table 2 presents the medical data of the study participants. A total of 94 (31.2%) patients were diabetic, 64 (21.3%) were hypertensive, while 143 (47.5%) were both diabetic and hypertensive. Among hypertensive patients, 72 (23.9%) had the disease for less than five years, while 74 (24.6%) had it for more than 10 years. Also, 91 (30.2%) had been diabetic for less than five years, and 99 (32.9%) had been for more than 10 years. CKD was diagnosed among 71 (23.6%) patients; 41 (13.6%) had it for less than five years while 11 (3.7%) had it for more than 10 years. Of note, 115 (38.2%) patients had a family history of CKD.

**Table 2:** Medical data of the study participants (N=301) CKD: chronic kidney disease; DM: diabetes mellitus; HTN: hypertension

Variables	Frequency (n)	Percent (%)
Patient disease		
HTN	64	21.3%
DM	94	31.2%
Both	143	47.5%
Duration of hypertension		
No hypertension	94	31.2%
<5 years	72	23.9%
6-10 years	61	20.3%
>10 years	74	24.6%
Duration of DM		
No DM	64	21.3%
<5 years	91	30.2%
6-10 years	47	15.6%
>10 years	99	32.9%
Duration of CKD		
No CKD	230	76.4%
<5 years	41	13.6%
6-10 years	19	6.3%
> 10 years	11	3.7%
Family history of kidney disease		
Yes	115	38.2%
No	186	61.8%

Table 3 details awareness of CKD among DM and hypertensive patients in the Aseer region. Of note, 276 (91.7%) study participants knew that kidney failure was fatal if not treated by dialysis or kidney transplant; 275 (91.4%) were aware that CKD reduces the ability of the kidneys to clear wastes from the blood; 271 (90%) reported that CKD could progress to kidney failure; 259 (86%) stated that Kidney failure treatment costs more than kidney function screening; and 245 (81.4%) knew that DM can cause CKD, while 233 (77.4%) reported that high blood pressure can lead to CKD. Significantly, 216 (71.8%) were aware that CKD may not manifest any symptoms until it reached an advanced stage.

**Table 3:** Awareness of CKD among the study population (N=301) CKD: chronic kidney disease; DM: diabetes mellitus

Awareness-related items	Yes		No	
	Frequency (n)	Percent (%)	Frequency (n)	Percent (%)
CKD reduces the ability of the kidneys to clear wastes from the blood	275	91.4%	26	8.6%
CKD may not have any symptoms until advanced	216	71.8%	85	28.2%
High blood pressure can cause CKD	233	77.4%	68	22.6%
DM can cause CKD	245	81.4%	56	18.6%
CKD could progress to kidney failure	271	90.0%	30	10.0%
Kidney failure is fatal if not treated by dialysis or a kidney transplant	276	91.7%	25	8.3%
Kidney failure treatment costs more than kidney function screening	259	86.0%	42	14.0%

**Figure 1:**
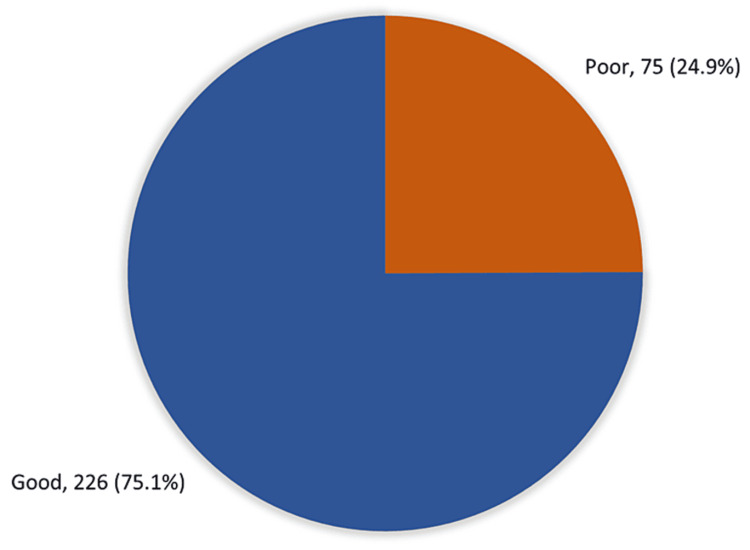
Overall level of awareness of CKD among the study population (N=301) CKD: chronic kidney disease

Figure 1 illustrates the overall awareness of CKD among DM and hypertensive patients in the Aseer region; 226 (75.1%) study participants had good awareness overall about CKD while only 75 (24.9%) reported a poor awareness level.

Table 4 summarizes attitudes and perceptions regarding CKD among DM and hypertensive patients in the Aseer region. A total of 285 (94.7%) study participants stated that they would go to a health facility if they had signs of kidney disease; 276 (91.7%) agreed that detection of CKD is important to slow its progress; and 271 (90%) were aware that CKD carries a high risk of death. Also, 267 (88.7%) thought that it is possible to prevent CKD, and 257 (85.4%) agreed that a kidney function test is necessary even if there are no signs of CKD. Only 207 (68.8%) reported that their blood pressure was controlled and within the normal range, and 209 (69.4%) stated that their blood sugar was controlled and within the normal range.

**Table 4:** Attitudes and perceptions regarding CKD among the study population (N=301) CKD: chronic kidney disease

Attitude-related items	Disagree		Agree	
	Frequency (n)	Percent (%)	Frequency (n)	Percent (%)
My blood sugar is controlled and within normal range	92	30.6%	209	69.4%
My blood pressure is controlled and within normal range	94	31.2%	207	68.8%
A kidney function test is necessary even if no sign of CKD exists	44	14.6%	257	85.4%
I do the annual routine kidney screening test	97	32.2%	204	67.8%
It is not too expensive to have a kidney screening test	88	29.2%	213	70.8%
CKD carries a high risk of death	30	10.0%	271	90.0%
I will go to a health facility if I have signs of kidney disease	16	5.3%	285	94.7%
It is possible to prevent CKD	34	11.3%	267	88.7%
Early detection of CKD is important to slow its progress	25	8.3%	276	91.7%

Table 5 details the factors associated with patients' awareness of CKD, in the Aseer region, Saudi Arabia; 138 (79.3%) of young patients had a good awareness level compared to 88 (69.3%) of older patients, and the difference was statistically significant (p=0.047). Also, 116 (80%) of highly educated patients had good knowledge levels compared to 37 (57.8%) of those with low education (p=0.002). Good knowledge was observed in 50 (82%) patients with hypertension for 6-10 years vs. 45 (60.8%) of those with the condition for more than 10 years (p=0.011). Likewise, 79 (86.8%) patients with DM for less than five years had good awareness in comparison to 45 (70.3%) of those without (p=0.017). Good knowledge was noted in 35 (85.4%) of patients with CKD for less than five years compared to five (45.5%) of those with the disease for more than 10 years (p=0.047). Of note, 97 (84.3%) patients with a family history of CKD had good awareness about the disease vs. 129 (69.4%) of those without (p=0.003).

**Table 5:** Factors associated with the awareness of CKD among the study population (N=301) *The p-values were determined using Pearson’s chi-squared test, with statistical significance set at p<0.05. ^$^Exact probability test CKD: chronic kidney disease; DM: diabetes mellitus; HTN: hypertension

Factors	Overall awareness level				P-value
	Poor		Good		
	Frequency (n)	Percent (%)	Frequency (n)	Percent (%)	
Age in years					0.047*
<55	36	20.7%	138	79.3%	
>55	39	30.7%	88	69.3%	
Gender					0.605
Male	48	24.0%	152	76.0%	
Female	27	26.7%	74	73.3%	
Educational level					0.002*
Below high school	27	42.2%	37	57.8%	
High school	19	20.7%	73	79.3%	
University/above	29	20.0%	116	80.0%	
Disease group					0.569
HTN	19	29.7%	45	70.3%	
DM	21	22.3%	73	77.7%	
Both	35	24.5%	108	75.5%	
Duration of HTN					0.011*
No HTN	21	22.3%	73	77.7%	
<5 years	14	19.4%	58	80.6%	
6-10 years	11	18.0%	50	82.0%	
>10 years	29	39.2%	45	60.8%	
Duration of DM					0.017*
No DM	19	29.7%	45	70.3%	
<5 years	12	13.2%	79	86.8%	
6-10 years	16	34.0%	31	66.0%	
>10 years	28	28.3%	71	71.7%	
Duration of CKD					0.047*^$^
No CKD	57	24.8%	173	75.2%	
<5 years	6	14.6%	35	85.4%	
6-10 years	6	31.6%	13	68.4%	
>10 years	6	54.5%	5	45.5%	
Family history of Kidney disease					0.003*
Yes	18	15.7%	97	84.3%	
No	57	30.6%	129	69.4%	

## Discussion

CKD is characterized by kidney damage and the gradual progression toward the organs' inability to clean the blood as well as healthy kidneys. This leads to toxic waste and extra fluid accumulating in the body and may result in high blood pressure, heart disease, stroke, and early death [14]. However, people with CKD and those at risk for the condition can take steps to protect their kidneys with the help of their healthcare providers and gain awareness of the features of the disease, its risk factors, and preventive measures. The current study aimed to assess the level of awareness, prevalence, and risk factors of CKD in diabetic and hypertensive patients. Concerning CKD prevalence, less than one-fourth of the study participants had the condition, and most of them had been recently diagnosed (less than five years). Our review of the literature showed that CKD is more prevalent among persons aged 65 years or older, ranging from 23.4 to 35.8%, while its prevalence among those 30 years or older is also significant (7.2%) [15]. Diabetes and HTN are the major causes of CKD in both the developed and developing world [16]. Type 2 DM (T2DM) is a major risk factor for CKD [17], and several studies have analyzed its role as an independent risk factor in the incidence of CKD [18,19,20].

According to the annual reports of the United States Renal Data System, nearly 25% of CKD cases could be attributed to HTN in 2013 [21,22,23]. van der Meer et al. [24] have reported that the prevalence of CKD was 28% in diabetes and 21% in HTN, which aligns with our findings. The presence of diabetes has been independently associated with albuminuria with normal glomerular filtration rate (eGFR). Also, Flores et al. [25] have documented that CKD was seen in 42.9% of diabetic patients, 21% of HTN patients, and 23.3% of hyperuricemia patients. Wang et al. [26] in their prospective study found that the prevalence of new-onset CKD in the group of HTN and diabetes patients was 27.1, and 25.4 per 1000 years, respectively. A study conducted in Saudi Arabia about diabetic-related kidney disease revealed that 43% of the patients had microalbuminuria, 19% had macroalbuminuria, and 37% had end-stage renal disease (ESRD) [27]. Another study has shown that the general prevalence of diabetic nephropathy was 10.8%, classified as follows: 1.2% microalbuminuria, 8.1% macroalbuminuria, and 1.5% ESRD [19]. In a study involving diabetic outpatient clinics, 12.8% of patients had dipstick proteinuria, and of the remaining patients, 41.3% had microalbuminuria [28].

Regarding patient awareness of CKD, our findings showed that most of the patients (three-fourths) were knowledgeable regarding the disease. to elaborate, most of the patients knew that CKD reduces the ability of the kidneys to clear waste from the blood; they also knew that CKD could progress to kidney failure and that kidney failure treatment costs more than kidney function screening. More than three-fourths knew that DM and high blood pressure can cause CKD. However, less than three-fourths knew that CKD may not manifest any symptoms until it reaches an advanced stage. Higher awareness was associated with younger age, high education, being hypertensive or diabetic, presence of CKD, and a family history of CKD. A lower level of awareness was found among patients in Ethiopia, where only 28.4% of the participants were aware of CKD and its risk factors [2]. In Nigeria, only 27% of study participants had good knowledge [29], but a higher level was reported in Jordan as 50% of the participants scored >80% in terms of correct answers [30].

A study conducted in Saudi Arabia by Nahlah Fahad et al. showed that the level of knowledge about CKD among diabetic patients was poor, moderate, and good among 46.8%, 29.2%, and 24%, respectively [31]. Another study conducted in Palestine reported that approximately half (46.8%) of the participants had a poor level of awareness regarding CKD. A moderate level of awareness was found in 29.3% of the participants, while 24% demonstrated a good level of awareness. Several factors were found to be associated with a higher level of awareness, such as having a bachelor's degree, being unmarried, being a student, and receiving information about CKD from a medical practitioner. Additionally, in the same study, it was noted that hypertensive patients in Palestine exhibited higher levels of knowledge and a more positive attitude toward the prevention and early detection of chronic renal disease [32].

Concerning patient attitudes, we found a generally positive attitude toward CKD and its effects and prevention. Another study conducted in Saudi Arabia has also reported a predominantly positive attitude about the prevention and control of CKD in the participants, in addition to a relatively high knowledge regarding the prevention of kidney patients among the educated Saudi population; the study recommended making greater efforts to enhance knowledge related to healthcare [33]. In general, one of the early steps that can be taken to determine the individual's ability to follow health-related behaviors is to assess knowledge, attitudes, and practices, as per the studies reviewed, as examining clinical indicators is essential for the early detection of patients at risk of developing CKD [34-35].

The study has several limitations. Firstly, the cross-sectional design employed in the study precluded establishing a definitive cause-and-effect relationship between sociodemographic factors and patient awareness about CKD. Moreover, it is important to note that the study was conducted in a specific region, and caution should be exercised when generalizing the findings to other regions in Saudi Arabia. Additionally, relying on self-reported data may have introduced an element of biases, such as social desirability or recall bias, which could impact the accuracy of the results. Lastly, the study did not directly observe participants' actual behaviors in terms of seeking medical care or adhering to recommendations.

## Conclusions

Based on our findings, CKD was not common among the study participants, and its prevalence in our cohort was found to be less than estimated based on studies in the literature. This may be attributed to the subjective assessment of the disease among our sample (instead of lab-based diagnosis). Also, diabetic and hypertensive patients showed a more than satisfactory level of awareness of CKD, especially younger patients with high levels of education. It is important to raise awareness among patients about various aspects of CKD so that they can modify their lifestyles to prevent its occurrence, as this can have a positive role in patients' understanding of the disease and its medical consequences.

## Appendices

**Table 6:** Questionnaire used in the study CKD: chronic kidney disease; DM: diabetes mellitus; HTN: hypertension

Section I: sociodemographic characteristics		
1) Age in years (Table 1)		
<55		
>55		
2) Gender (Table 1)		
Male		
Female		
3) Educational level (Table 1)		
Below high school		
High school		
University/above		
4) Duration of HTN (Table 2)		
No HTN		
<5 years		
6-10 years		
>10 years		
5) Duration of DM (Table 2)		
No DM		
<5 years		
6-10 years		
>10 years		
6) Duration of CKD (Table 2)		
No CKD		
<5 years		
6-10 years		
>10 years		
7) Family history of Kidney disease (Table 2)		
Yes		
No		
Section II: knowledge-related questions		
8) CKD reduces the ability of the kidneys to clear wastes from the blood (Table 3)	Yes	No
9) CKD may not have any symptoms until advanced (Table 3)	Yes	No
10) High blood pressure can cause CKD (Table 3)	Yes	No
11) DM can cause CKD (Table 3)	Yes	No
12) CKD could progress to kidney failure (Table 3)	Yes	No
13) Kidney failure is fatal if not treated by dialysis or kidney transplant (Table 3)	Yes	No
14) Kidney failure treatment costs more than kidney function screening (Table 3)	Yes	No
Section III: attitude-related questions		
15) My blood sugar is controlled and within normal range (Table 4)	Disagree	Agree
16) My blood pressure is controlled and within normal range (Table 4)	Disagree	Agree
17) A kidney function test is necessary even if there is no sign of CKD (Table 4)	Disagree	Agree
18) I do the annual routine kidney screening test (Table 4)	Disagree	Agree
19) It is not too expensive to have a kidney screening test (Table 4)	Disagree	Agree
20) CKD carries a high risk of death (Table 4)	Disagree	Agree
21) I will go to a health facility if I have signs of kidney disease (Table 4)	Disagree	Agree
22) It is possible to prevent CKD (Table 4)	Disagree	Agree
23) Early detection of CKD is important to slow its progress (Table 4)	Disagree	Agree
